# Quality indicators in colonoscopies performed in digestive endoscopy services in northeast Brazil

**DOI:** 10.1590/0102-672020260000045e1974

**Published:** 2026-10-05

**Authors:** Anna Isabel Rodrigues ALVES, Fábio Gomes TEIXEIRA

**Affiliations:** 1 Universidade Federal do Maranhão, University Hospital, Digestive Endoscopy Service – São Luis (MA), Brazil.

**Keywords:** Colonoscopy, Colorectal Neoplasms, Polyps, Adenoma, Mass Screening, Colonoscopia, Neoplasias Colorretais, Pólipos, Adenoma, Programas de Rastreamento

## Abstract

**Background:**

Colorectal cancer is the third most frequently diagnosed cancer worldwide. Colonoscopy is fundamental in reducing its incidence and mortality, provided it follows certain quality parameters. Examples of these quality parameters are the cecal intubation rate, adenoma detection rate (ADT), and the adequate percentage of patients with satisfactory bowel preparation.

**Aims:**

To measure the quality parameters of a colonoscopy service in Northeast Brazil. Methods: A cross-sectional, retrospective study, with data collected from electronic medical records, including screening colonoscopies between 2022 and 2024.

**Results:**

565 colonoscopies were included in the study. The overall ADR was 24.78, 21.27% in women and 32.03% in men. The cecal intubation rate was approximately 95%, and most patients (91.3%) had excellent bowel preparation. The polyp detection rate was 39.47%.

**Conclusions:**

The cecal intubation rate and the proportion of patients with adequate bowel preparation were satisfactory. However, the polyp detection rate was lower than recommended in the literature. Thus, evaluating these parameters is a way to monitor the quality of colorectal cancer prevention, improving the strategies employed, even in the face of the limitations encountered.

## ARTICLE HIGHLIGHTS

The study assessed the quality of a colorectal cancer (CRC) screening program at a colonoscopy service located in a city in northeastern Brazil, from 2022 to 2024.

The results indicate that the service meets the quality parameters related to the cecal intubation rate and bowel preparation quality.

The adenoma detection rate, which is the primary quality parameter, meets the European guidelines but falls below the current recommendations of the American task force.

Sample limitations, such as the study being conducted at a single center and the predominant use of low-definition equipment, may explain the findings, and monitoring these indicators is essential to improve local CRC prevention strategies.

## Summary

**CENTRAL MESSAGE:**

Colorectal cancer (CRC) is the third most common cancer worldwide, with high morbidity and mortality. There is substantial evidence supporting the role of colonoscopies in reducing the incidence and mortality of this cancer, due to its impact on the identification and removal of precancerous lesions. However, this effect depends on the performance of high-quality colonoscopies. There are several priority quality indicators for colonoscopy examinations, such as the adenoma detection rate (ADR), the rate of adherence to recommended screening and surveillance intervals, the rate of adequate bowel preparation, and the cecal intubation rate. Achieving these quality indicators is closely associated with a reduction in interval CRC (I-CRC), which can be defined as the CRC that develops following a negative screening examination.

**PERSPECTIVES:**

In this study, the cecal intubation rate and the percentage of examinations with adequate bowel preparation comply with the most current recommendations. Considering screening colonoscopies and the recommendations of the European Society of Gastrointestinal Endoscopy (ESGE), the adenoma detection rate is also adequate. Conversely, the polyp detection rate was lower than that recommended in the literature. Furthermore, when considering the most recent recommendations of the American task force (ASGE/ACG), the adenoma detection rate was also below the expected level. To improve these parameters, a set of measures involving pre-procedure actions, details regarding technique, and tools to assist with the examinations should be considered. Thus, conducting studies aimed at analyzing the quality of screening programs is a means of promoting critical self-assessment and advancing the strategies currently employed.

## VISUAL ABSTRACT

**Figure:**
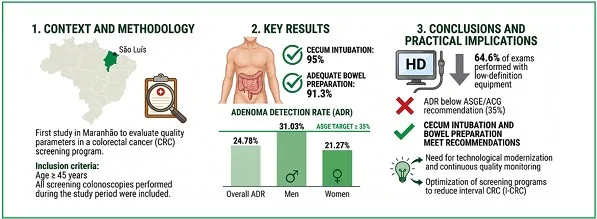

## RESUMO

**Racional:**

O câncer colorretal é o terceiro câncer mais diagnosticado no mundo. A colonoscopia é fundamental na redução da sua incidência e mortalidade, desde que siga alguns parâmetros de qualidade. Exemplos desses parâmetros de qualidade são a taxa de intubação cecal, taxa de detecção de adenomas (TDA) e porcentagem adequada de pacientes com preparo intestinal satisfatório.

**Objetivos:**

Mensurar os parâmetros de qualidade de um serviço de colonoscopia no Nordeste do Brasil. Métodos: Estudo transversal, retrospectivo, com coletas de dados em prontuário eletrônico, compreendendo colonoscopias de rastreio entre 2022 e 2024.

**Resultados:**

565 colonoscopias foram incluídas no estudo. A TDA geral foi de 24,78%, sendo 21,27% em mulheres e 32,03% em homens. A taxa de intubação cecal ficou em torno de 95% e a maior parte dos pacientes (91,3%) tiveram um preparo intestinal excelente. A taxa de detecção de pólipos foi de 39,47%.

**Conclusões:**

A taxa de intubação cecal e a porcentagem de pacientes com preparo intestinal adequado foi satisfatória. Por outro lado, a taxa de detecção de pólipos foi inferior ao recomendado em literatura. Dessa forma, avaliar esses parâmetros é uma forma de monitorar a qualidade da prevenção ao câncer colorretal e aprimorar as estratégias utilizadas, mesmo diante das limitações encontradas.

## INTRODUCTION

 Colorectal cancer (CRC) is the third most common cancer worldwide, with high morbidity and mortality. An estimated 45,630 new cases occur annually in Brazil, ranking fourth in terms of incidence in the Northeast region (13.08 per 100,000 inhabitants). In the state of Maranhão, an estimated 520 cases were projected for 2023. For the same year, in São Luís, the state capital, the estimate was 160 new cases. Regarding mortality, 20,245 deaths occurred in Brazil in 2020 (9.56 per 100,000 inhabitants)^19,20^. 

 There is substantial evidence supporting the role of colonoscopies in reducing the incidence and mortality of this cancer, due to its impact on the identification and removal of precancerous lesions. However, this effect depends on the performance of a high-quality colonoscopy^21^. There are several priority quality indicators for colonoscopy examinations, such as the adenoma detection rate (ADR), the rate of adherence to recommended screening and surveillance intervals, the rate of adequate bowel preparation, and the cecal intubation rate^6,22^. 

 Achieving these quality indicators is closely associated with a reduction in interval CRC (I-CRC), which can be defined as the CRC that develops after a negative screening examination^2^. Among these parameters, ADR is the most clinically relevant and best-validated quality indicator in colonoscopy. It is based on the percentage of patients with at least one conventional adenoma detected and confirmed by pathology during a screening colonoscopy. ADR targets are defined as an average of 35% (40% for men and 30% for women)^22^. 

 Considering the importance of measuring the quality parameters of a colonoscopy service in reducing interval CRC (I-CRC), and taking into account the scarcity of data on this subject in several regions of the country, the current study was conducted to assess selected quality parameters in a colonoscopy service in northeastern Brazil. Thus, it will be possible to evaluate the quality and impact of this service within the colorectal cancer prevention pathway. 

## METHODS

 This was a cross-sectional, retrospective study involving data collection from electronic medical records. The data collected referred to screening colonoscopy examinations performed between the years of 2022 and 2024. The study was conducted at a private-sector clinic in the city of São Luís, Maranhão. This facility is a referral center for colonoscopy examinations and provides care to patients from throughout the state of Maranhão. The examinations included in this study were performed by a single examiner with experience in conducting colonoscopies. The study was approved by the Institution’s Research Ethics Committee (CAAE number 7.363.705). 

## RESULTS

 Between January 2022 and December 2024, 836 colonoscopies were performed at the studied private-sector service facility. Of these examinations, 271 were excluded as they did not meet the inclusion criteria or due to the electronic medical records containing insufficient data for analysis. Therefore, 565 screening colonoscopies were analyzed in this study. 

 The sample was predominantly female (n=362; 64%), with a mean age of approximately 60 years. It was observed that 96.28% of the patients undergoing the examination were sedated by the endoscopists themselves, and the most frequently used device was the Pentax© EPK-1000 (n=365; 64.6%). In most examinations, the cecum (n=535; 94.69%) and ileum were reached (n=502; 88.85%). Rectal retroflexion was performed in 434 examinations (76.81%), and most examinations (80.88%; n=457) showed excellent bowel preparation quality (Table 1). 

**Table 1:** Variables analyzed in screening colonoscopies performed between January 2022 and December 2024 at an endoscopy center in the state of Maranhão.

Variables		n (%)
Age	Male	59.84
	Female	59.99
Sex	Male	203 (35.93)
	Female	362 (64.07)
Sedation	Endoscopist	544 (96.28)
	Anestesiologist	21 (3.72)
Device used	Olympus CLV-190 ®	200 (35.4)
	Pentax EPK-1000 ®	365 (64.6)
Cecal intubation	Yes	535 (94.69)
	No	30 (5.31)
Ileal intubation	Yes	502 (88.85)
	No	63 (11.15)
Rectal retroflexion	Yes	434 (76.81)
	No	131 (23.19)
Presence of polyps	Yes	223 (39.47)
	No	342 (60.53)
Polyp detection rate by sex	Female	97 (26.79)
	Male	126 (62.06)
Adenoma detection	Yes	140 (24.78)
	No	425 (75.22)
Adenoma detection rate by sex	Female	77 (21.27)
	Male	63 (31.03)
Bowel preparation quality	Inadequate	31 (5.49)
	Poor	18 (3.19)
	Good	59 (10.44)
	Excellent	457 (80.88)

 The polyp detection rate was 39.47%. Regarding the adenoma detection rate, the value found was approximately 25%. Considering the female subgroup, this latter variable was 21.27%. In male patients, the adenoma detection rate was 31.03% (Table 1). 

 Regarding patients diagnosed with adenoma, the following characteristics were observed (Table 2): most underwent colonoscopy using the Pentax EPK-1000 device (n=130; 93.29%), had cecal intubation (n=134; 95%), excellent bowel preparation (n=116; 82.8%), and underwent rectal retroflexion (n=101; 72%). Regarding location, adenomas were most frequently found in the transverse colon (32.1%), ascending colon (27.1%), sigmoid colon (14.2%), cecum (14.28%), descending colon (6.42%), rectum (4.38%), and ileocecal valve (1.46%). 

**Table 2:** Characteristics of patients diagnosed with adenoma in screening colonoscopies performed between January 2022 and December 2024 at an endoscopy center in the state of Maranhão.

Variables in patients with adenoma		n (%)
Device used	Olympus CLV-190 ®	10 (6.71)
	Pentax EPK-1000 ®	130 (93.29)
Cecal intubation	Yes	134 (95.71)
	No	6 (4.28)
Rectal retroflexion	Yes	101 (72.14)
	No	39 (27.86)
Bowel preparation quality	Inadequate	7 (5)
	Poor	4 (2.86)
	Good	13 (9.28)
	Excellent	116 (82.86)
Location	Ascending colon	38 (27.14)
	Cecum	20 (14.28)
	Descending colon	9 (6.42)
	Rectum	6 (4.28)
	Sigmoid colon	20 (14.28)
	Transverse colon	45 (32.14)
	Ileocecal valve	2 (1.46)

## DISCUSSION

 In the analysis of the epidemiological profile of the patients enrolled in the screening program evaluated in this study, females were the most prevalent, and the mean age was approximately 60 years. Although women predominated in this sample, most patients diagnosed with polyps and adenomas were male. Considering the role of diagnosing these lesions in the development of CRC, these data are consistent with what has been described in the current literature: CRC-related incidence and mortality rates are higher among male patients, including in the state of Maranhão^10,12^. 

 There are several explanations for these differences. Studies have highlighted the role of lifestyle habits, dietary intake, alcohol consumption, the incidence of obesity, and physical inactivity among men. Another explanation is a worldwide trend toward greater adherence by women to CRC screening programs, leading to the earlier diagnosis and removal of precursor lesions. These data reinforce the importance of identifying the most vulnerable populations and thereby directing actions that promote awareness, detection, and the reduction of morbidity and mortality rates associated with the disease^10,12^. 

 One explanation for the differences in the values found is that the new ASGE/ACG Task Force recommendation now includes other indications in the calculation of ADR, whereas the ESGE includes only screening colonoscopies, as was the case in our sample. This new recommendation suggests that screening examinations, surveillance examinations, and diagnostic colonoscopies following positive non-fecal tests should be included. In addition, studies conducted over recent decades that have indicated that steady increases in ADR were taken into account^22^. However, these studies were conducted in the United States and reflect a setting in which high-definition colonoscopes are widely available, unlike what is offered in other parts of the world. 

 Comparing the ADR values in our sample with studies conducted in other regions of the country, considerable heterogeneity was observed. In another study conducted in the Northeast region, the mean value found was 16%^17^. In the Central-West region, the rate found was 27.1%^4^. Studies conducted in the Southeast region found values ranging from 30.8 to 40.5%^14,15^. In the South region, the values ranged from 26.5 to 37.3%^11,23^. 

 These data suggest that shortcomings in CRC screening programs persist in many regions of the country, whether because of limited access within the Brazilian Unified Health System or low adherence within the supplementary healthcare system. Other factors may also be considered, such as cultural and educational issues among the population. However, the variability of the results reinforces the need for multicenter studies that can more clearly establish the Brazilian reality^3,9,17^. 

 The importance of ADR is already well established in the literature. A study involving 45,026 individuals from the Polish National Colorectal Cancer Screening Program found a 10-fold higher risk of I-CRC when comparing endoscopists with an ADR below 20% with those with an ADR of 20% or higher^21^. In addition, Corley et al.^8^, when analyzing 314,872 colonoscopies, demonstrated that a 1% increase in ADR was associated with a 3% reduction in the risk of I-CRC. 

 Despite the relevance of this quality parameter, it is important to highlight some limitations inherent to it. Endoscopists may take advantage of identifying and removing a single polyp while neglecting the careful examination of the remainder of the colonic mucosa. Thus, even with adequate adenoma detection rates, an endoscopist may still miss potentially malignant lesions^18^. To mitigate this gap related to the interpretation of the measure, another suggested parameter is adenomas per colonoscopy (APC). In this parameter, the total number of adenomas detected is divided by the total number of examinations. Most data suggest that an APC of approximately 0.5–0.6 is consistent with the current minimum ADR thresholds^1,6,7,21^. 

 Another quality metric assessed was the polyp detection rate (PDR). In our sample, the achieved rate was 39.47%, similar to the 36.6% described in a multicenter study conducted in the state of São Paulo^15^. These values are below the rate of at least 42.7% previously described in the literature as contributing to a reduction in the relative risk of interval CRC^24^. 

 Another characteristic observed in our sample was that most examinations were performed using a low-definition device (n=365; 64.6%). A study conducted by the Universidade de São Paulo found a statistically significant association between the use of high-definition endoscopes and an increased polyp detection rate^15^. This parameter may be one of the explanations for the ADR and PDR values found below those recommended in the current literature. 

 Regarding the other quality parameters studied, a cecal intubation rate of approximately 95% was obtained. Reaching this intestinal segment ensures a complete examination of the colon and reduces the risk of interval CRC (I-CRC). Recent recommendations suggest that cecal intubation should be achieved in ≥90% of all colonoscopies and ≥95% of screening colonoscopies^5,21^. Hsu and Chiu^13^ reported a higher risk of I-CRC for colonoscopies that were incomplete within a screening program. 

 Adequate bowel preparation was found in 91.3% of the sample. This quality parameter is basic and fundamental. Several studies and meta-analyses have demonstrated that inadequate bowel preparation significantly reduces adenoma detection. In addition, poor preparation may lead to prolonged procedures, repeated colonoscopies, and increased costs^5,13,16^. The current recommendation is that at least 90% of patients undergoing colonoscopy should have satisfactory preparation, defined by the description of the preparation as excellent, good, or adequate^22^. 

 This is the first study conducted in the state of Maranhão to evaluate quality parameters in a colorectal cancer screening program. Since the study was restricted to a single facility, the results may not accurately reflect the reality of screening programs in the state. The findings that were inadequate may be related to the limitations of the sample, such as the use of low-definition devices in most patients, which is known affect the reduction of interval colorectal cancer^22^. 

 Overall, improvements in quality parameters, particularly ADR, should always be pursued to optimize screening programs. The use of high-definition colonoscopes, whose resources may be enhanced with artificial intelligence tools, in addition to devices such as Endocuff and a cap/hood placed at the tip of the device, assists in performing the examinations^21,24^. In settings where these resources are unavailable, as was the case in this sample, high-quality scope withdrawal should be pursued, involving: adequate preparation, washing and aspiration, distension and exposure of the entire mucosa, as well as systematic re-examination of the right colon. In addition, it is important to train colonoscopists who are qualified and committed to screening programs. One potential improvement would be to include the new ASGE/ACG recommendations in the calculation of ADR in future studies^22^. 

## CONCLUSIONS

 In this study, the cecal intubation rate and the percentage of examinations with adequate bowel preparation comply with the most current recommendations. Considering screening colonoscopies and the ESGE recommendation, our adenoma detection rate is also adequate. However, according to the ASGE/ACG Task Force recommendation, our ADR is below the recommended level. The polyp detection rate was also lower than that recommended in the literature. To improve these parameters, a set of measures involving pre-procedure actions, details regarding technique, and tools to assist with the examinations should be considered. Therefore, conducting studies aimed at analyzing the quality of screening programs is a means of promoting critical self-assessment and advancing the strategies currently employed. 

## Data Availability

The datasets generated and/or analyzed during the current study are available from the corresponding author upon reasonable request.
